# Integration of Carbon Nanotubes in an HFCVD Diamond Synthesis Process in a Methane-Rich H_2_/CH_4_ Gas Mixture

**DOI:** 10.3390/ma16206755

**Published:** 2023-10-19

**Authors:** Alexander Mitulinsky, Alexander Gaydaychuk, Sergei Zenkin, Stanislav Meisner, Vlada Bulakh, Stepan Linnik

**Affiliations:** 1 National Research Tomsk Polytechnic University, 634050 Tomsk, Russia; gaydaychuk@tpu.ru (A.G.); zen@tpu.ru (S.Z.); vladabulakh@tpu.ru (V.B.); linniksa@tpu.ru (S.L.); 2 Institute of Strength Physics and Materials Science SB RAS, 634055 Tomsk, Russia; msn@ispms.tsc.ru

**Keywords:** HFCVD, carbon nanotubes, hydrogen etching

## Abstract

In this work, we present experimental data on carbon nanotubes integration during diamond synthesis. Carbon nanotubes layers were preliminarily deposited on silicon and diamond substrates, after which the substrates were loaded into the HFCVD reactor for further growth of the diamond phase. The CVD process was held in an argon-free H_2_/CH_4_ working gas mixture without the use of a catalyst for carbon nanotubes growth. It is shown that in a wide range of studied working gas composition (CH_4_ concentration up to 28.6 vol.%) nanotubes etched from the substrate surface before the diamond growth process began.

## 1. Introduction

Carbon nanotubes (CNT) are an outstanding material combining great mechanical, electrical and thermal properties [1,2,3,4,5,6,7]. CNT is the material of choice for the reinforcement of composite materials [8,9]. There are three main methods for the synthesis of CNTs: laser ablation, arc discharge and chemical vapor deposition (CVD) [10,11,12]. CVD is the most promising method, due to its ability to upgrade this technique to an industrial scale, the high purity of synthesized nanotubes, its high yield and relatively low cost [13]. The key feature of CVD synthesis of CNTs is that the metal catalyst particles should be involved, such as iron, nickel, cobalt or their mixtures. The most common working gas mixture for the synthesis of CNTs is H_2_/CH_4_, and there are also reports on using the addition of argon (C_2_H_2_/Ar/H_2_) [14] and nitrogen (CH_4_/N_2_/H_2_) [15].

Another allotrope of carbon, diamond is widely used in industry as a protective coating for tools and mechanisms subject to abrasive wear [16,17]. Diamond coatings are also synthesized using the CVD method. Moreover, the deposition parameters of these materials are quite similar: a substrate temperature of 700–1000 °C for diamond [18] and 500–1200 °C for CNTs [13], chamber pressure of 10–100 Torr for diamond [18] and 10–750 Torr for CNTs [13] and CH_4_/H_2_ working gas mixtures of 1–30% CH_4_ for diamond and 3–80% for CNTs [19,20,21]. Despite the excellent physical and mechanical properties of diamond (high hardness, elastic modulus, wear resistance), the disadvantage of diamond coatings is their brittleness. A possible way to solve this problem is to create a diamond–CNTs composite. 

Recently, diamond–CNTs composites have received attention among researchers [21,22,23,24,25]. A material combining diamond and CNTs can exhibit interesting properties due to the synergy of these two materials, with improved mechanical, thermal and electrical properties. Possible benefits of diamond–CNTs composites have been reported or speculated for electrical, electronic and thermal applications (electrodes, field emission devices, MEMS/NEMS, thermionic energy generation, thermal interfaces) [24,25,26]. Moreover, the mechanical properties of diamond protective coatings can be improved by impregnating CNTs into their structure, which can increase the service life of diamond-coated tools and mechanisms.

In most of the works devoted to the combination of CNTs and diamond, these materials were grown simultaneously with the CVD method, using a window in the deposition parameters, with catalyst nanoparticles for CNTs growth. Table 1 presents the deposition parameters used in various studies of composite growth.

However, if catalyst nanoparticles are involved, it is only possible to synthesize a porous volume of diamond–CNTs material, while the growth of a dense nonporous diamond film with CNTs distributed in its volume is impossible, since the catalyst, upon interaction with diamond, causes its graphitization, which inevitably decreases the mechanical properties of the diamond film and significantly decreases the adhesion to the substrate [27,28].

One possible solution to avoid the problem of diamond graphitization is the pre-deposition of CNTs on the surface of the substrate and the subsequent growth of diamond over this layer. N. Shankar et al. [29] tested this approach with the following deposition parameters: HFCVD method, H_2_/CH_4_ atmosphere (1–5% CH_4_), substrate temperature 800 °C and chamber pressure 25 Torr. The authors report the etching of CNTs at 1% CH_4_, but with an increase in the concentration of methane to 2–5% they observed a window in the diamond phase growth parameter wherein CNTs were not destroyed. This reveals another problem of diamond–CNTs composite synthesis, which is H-etching of CNTs. F.-B. Rao et al. [30] treated CNTs at high temperatures in a thermal CVD reactor with pure hydrogen, and found that CNTs were preserved after hydrogen treatment at 800 °C, but were etched at 900 °C. The etching of CNTs was reported even at less harsh conditions. G. Zhang et al. [31] treated pre-deposited CNTs layer using RF-plasma in a hydrogen atmosphere (1 Torr) and temperatures from 200 to 400 °C. As a result, at all studied parameters they observed intensive CNTs etching.

In any case, thick (at least ~2 μm), dense and nonporous diamond–CNTs composite film has not yet been presented in the literature. Moreover, the conditions under which CNTs are preserved in a hydrogen environment remain unclear. The purpose of this work was to study further the processes occurring with pre-deposited CNTs layers in an HFCVD reactor under the conditions of diamond deposition at various methane amounts in the working mixture (5–28 vol.% CH_4_).

## 2. Materials and Methods

We used two types of substrates in this work:

(1) The mirror-polished single crystal Si (100) plates of 10 × 10 × 0.38 mm size;
(2) Microcrystalline diamond films grown on Si substrates, similar to those described above (film thickness ~5 μm).

Microcrystalline diamond films were grown with an HFCVD system. Prior to the diamond synthesis, substrates were ultrasonicated first in acetone, for 10 min, then in an aqueous solution of nano-diamond (diamond particle size 5–9 nm, mass fraction 0.035 wt.%) (FRPC “Altai”, Biysk, Russia) for 10 min, and again in acetone for 5 min. The substrate temperature during diamond synthesis was maintained at 850 ± 20 °C, and the operating pressure was maintained at 20 ± 1 Torr using a needle valve. The substrate to filament distance was 10 ± 1 mm. H_2_/CH_4_ (5.6 vol.% CH_4_) working gas composition was used for microcrystalline diamond synthesis with a total flow of 106 mL/min. Diamond film deposition time was 6 h.

**Figure 1:**
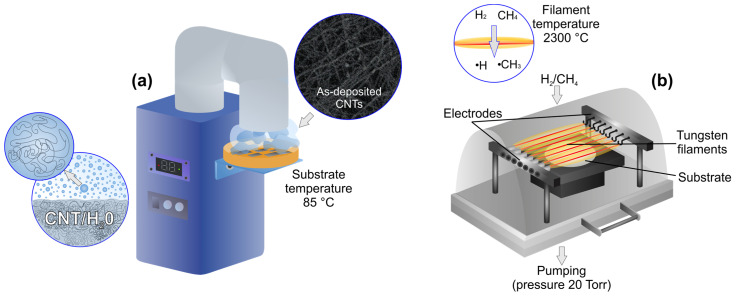
Carbon nanotube deposition setup (**a**); bubble inserts show schematic view of CNTs-containing liquid vaporization and SEM image of as-deposited CNTs layer; HFCVD setup (**b**); bubble insert shows cross-sectional view on hot filaments, explaining gas activation chemistry.

**Figure 2:**
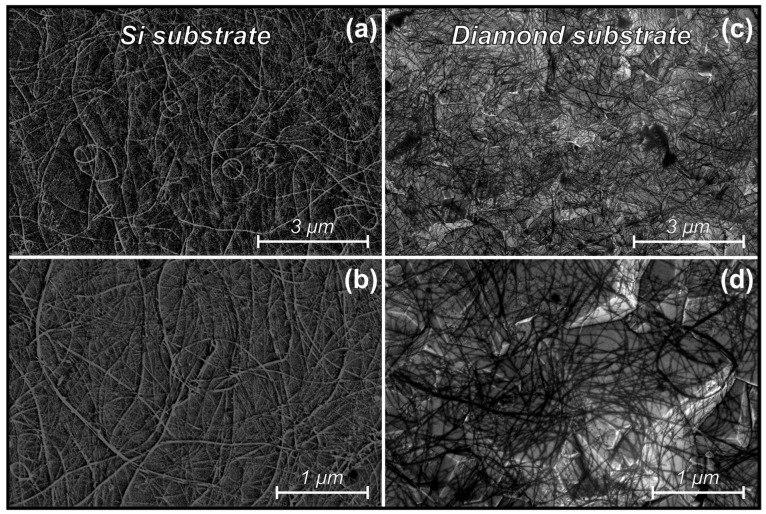
SEM images of as-deposited CNTs on Si (**a**,**b**) and diamond (**c**,**d**) substrates.

Prior to CNTs deposition, all substrates were ultrasonicated in acetone for 10 min. CNTs were deposited on a self-made installation, shown in Figure 1a. The reservoir of the CNTs deposition installation was equipped with an ultrasonic membrane. The reservoir was filled with a mixture of distilled water (98 vol.%) and a suspension of single-wall carbon nanotubes (SWCNT) (SWCNTs concentration in suspension 0.1%, carrier liquid H_2_O) (2 vol.%) TUBALL BATT (OCSiAl, Novosibirsk, Russia), which vaporized under ultrasonic action of the membrane. Next, CNTs-containing vapor flow was directed to the substrates, and placed on the substrate holder. The substrate holder was equipped with a heating system, and the temperature of the substrates was held at 85 ± 2 °C. The CNTs deposition time was 20 min. After CNTs’ layer deposition, substrates were treated with HFCVD (Figure 2b). The substrates were heated up to ~850 °C during the CVD treatment, the operating pressure was 20 ± 1 Torr and the substrate to filaments distance was 12 ± 1 mm. H_2_/CH_4_ mixture was used as a working gas, and the flow rate of hydrogen was held constant at 100 mL/min, while methane varied from 6 to 40 mL/min (5.6–28.6 vol.%).

Structure and morphology were investigated using an Apreo S LoVac (Thermo Fisher Scientific, Waltham, MA, USA) scanning electron microscope and JEM-2100F (JEOL, Tokyo, Japan) transmission electron microscope. Raman spectra were observed using a confocal Raman microscope coupled with the Scanning Probe Optical Unit NTEGRA Spectra (NT-MDT, Moscow, Russia). Cumulative mass loss (CML) data measurements were performed using microbalance MXA 21 (RADWAG, Radom, Poland). For CML measurements for each methane concentration, three samples were obtained.

The nanotube diameters were measured using ImageJ software. A sample of 40 measurements was taken to calculate the arithmetic average diameter. Since the measurement error of the nanotube diameter depends mainly on the SEM image resolution, it was constant and amounted to ±1.2 nm.

Samples were prepared for TEM using the carbon extraction replica method. First, a thin layer of amorphous carbon was deposited on the samples, and then gelatin was applied over the amorphous carbon layer. When dried, the gelatin tore off part of the coating from the sample surface. Next, the detached films were immersed in distilled water to dissolve the gelatin, after which they were dried and were ready for TEM analysis.

Raman ratios were calculated from the Raman mapping data as the arithmetic average of a sample of 10 spectra.

## 3. Results and Discussion

### 3.1. Characterization of As-Deposited CNTs Layer

Figure 2 shows the SEM images of as-deposited CNTs on silicon (a, b) and diamond substrates (c, d). The image settings were adjusted in order to improve the contrast of the CNTs, which may cause the CNTs to appear dark or bright in some images. SEM scanning reveals that the nanotube deposition method used in this work allows nanotubes to be deposited in a uniform layer over the entire surface of the samples. The CNTs deposition time was selected experimentally to ensure both that the structure of the CNTs web was sufficiently dense and that the substrate was not completely covered by a layer of nanotubes to allow diamond nucleation on the surface of the substrate. It is known that silicon is carbonized during CVD and forms a SiC surface layer [32,33], and there is a possibility of dissolution of carbon from CNTs in this process, so we used diamond substrates to find out if this process occurs or not.

**Figure 3:**
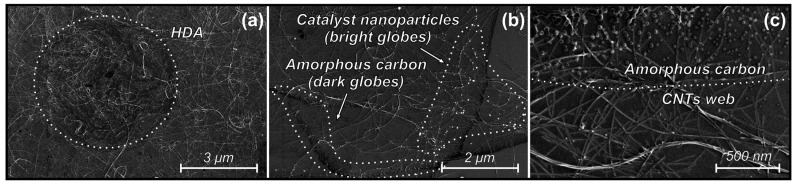
SEM images of high-density agglomerations of carbon nanotubes (**a**), catalytic nanoparticles presented in the sample (**b**) and amorphous carbon (**b**,**c**).

Figure 3 shows some of the features of the deposited nanotubes layer observed during the analysis of SEM images. In particular, agglomerations with a high packing density of nanotubes (HDA) were found (Figure 3a). The nanotube layer also contained residual catalyst nanoparticles observed as bright globes in Figure 3b, which were the feature of the CNTs suspension used. An insufficient amount of amorphous carbon can also be observed in the form of dark globes in Figure 3b,c.

The I_d_/I_g_ Raman peak ratio (Table 2) indicates the high quality and phase purity of carbon nanotubes.

### 3.2. SEM Observations

**Figure 4:**
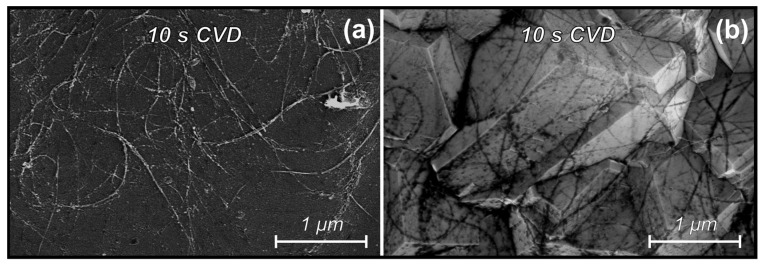
SEM images of CNTs after exposure to a H_2_/CH_4_ environment (5.6 vol.% CH_4_) for 10 s on Si (**a**) and diamond (**b**) substrates.

Figure 4 shows SEM images of CNTs after exposure to a H_2_/CH_4_ environment under the conditions of microcrystalline diamond (5.6 vol.% CH_4_) synthesis on Si (a) and diamond (b) substrates. In the case of the deposition of diamond and/or carbon nanotubes by the HFCVD method, an important role is played by atomic hydrogen, which is formed upon activation of the gas mixture with hot filaments, because it acts as an etchant. Interaction with such a high portion of atomic hydrogen in the gas mixture causes the rapid etching of nanotubes from the surface of the sample.

After 10 s of operation of the HFCVD (5.6 vol.% CH_4_) reactor, the density of the CNTs layer was highly reduced, and visible deformation and cutting of the tubes was observed. The calculated average diameter of as-deposited nanotubes was 27 nm, while after 10 s etching under such harsh conditions it decreased to 17 and 18 nm on Si and diamond substrates, respectively, and the largest CNTs diameter decreased from 45.1 ± 1.2 to 32.4 ± 1.2 and 35.2 ± 1.2 nm on Si and diamond substrates, respectively. This observation indicates the absence of a sufficient influence of substrate material on the CNTs etching, hence no carbonization of the Si substrate by CNTs.

This indicates, firstly, that the diameter of a nanotube significantly affects its etching time, and secondly, that the diameter of large nanotubes decreases during etching. It will be shown later in the TEM observations section that the visible “nanotube” is a bundle of nanotubes, and the thinning process is explained by the fact that nanotubes from the surface of the bundle are etched, while nanotubes inside the bundle are mostly preserved. Hydrogen etching also causes the cutting of nanotubes, which begins at defect sites [31]. Further, after 30 s of CVD exposure, the CNTs almost completely disappeared from the surface of the sample (not presented). The hydrogen etching tendency of CNTs was traced for all studied experimental conditions (CH_4_ concentration up to 28.6 vol.%).

**Figure 5:**
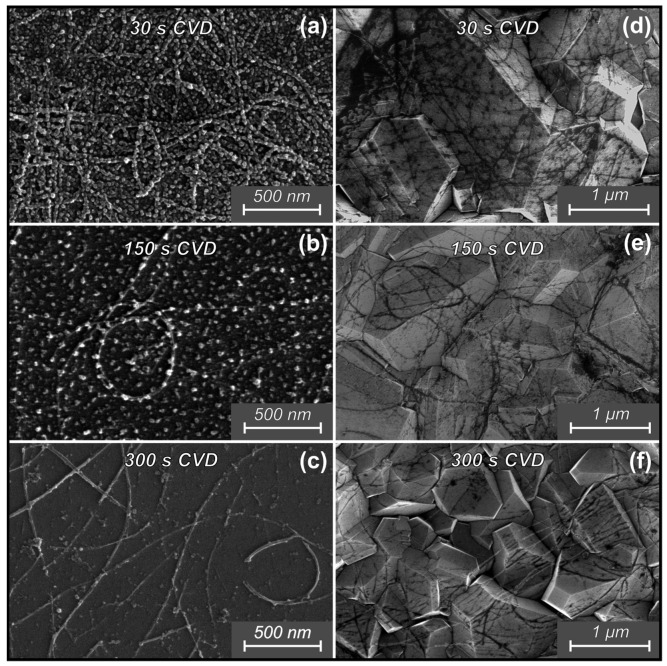
SEM images of CNTs after exposure to a H_2_/CH_4_ environment (28.6 vol.% CH_4_) on Si (**a**–**c**) and diamond (**d**–**f**) substrates.

Figure 5 shows SEM images of CNTs after exposure to a methane-rich H_2_/CH_4_ environment (28.6 vol.% CH_4_) on Si and diamond substrates.

After 30 s of operation of the HFCVD (28.6 vol.% CH_4_) reactor, the deposition of amorphous carbon in the form of globes on the surface of the substrates could be observed (Figure 5a,d). This is explained by the fact that with methane-rich gas composition and a short operating time (the reactor does not enter the stable operating mode), the low temperature of the substrate and the predominance of methyl radicals over atomic hydrogen in the activated gas mixture ensures the growth of the amorphous carbon phase and the low rate of H-etching of both CNTs and the grown amorphous carbon. The further etching of both nanotubes and amorphous carbon was observed after 150 s of CVD exposure (Figure 5b,e). The average CNTs diameter increased from 19 (for 30 s) to 24 nm, while the number of nanotubes was significantly reduced. The reason for the increase in the average diameter is that smaller CNTs bundles have lower volume, and hence significantly fewer nanotubes per bundle than larger bundles. The density of the amorphous carbon was significantly lower than that at 30 s. The results observed after 300 s of methane-rich environment treatment (Figure 5c,f) were close to those after 10 s in a 5.6 vol.% CH_4_ environment. The same defects were observed in the form of the thinning of the nanotube walls and cuts.

**Figure 6:**
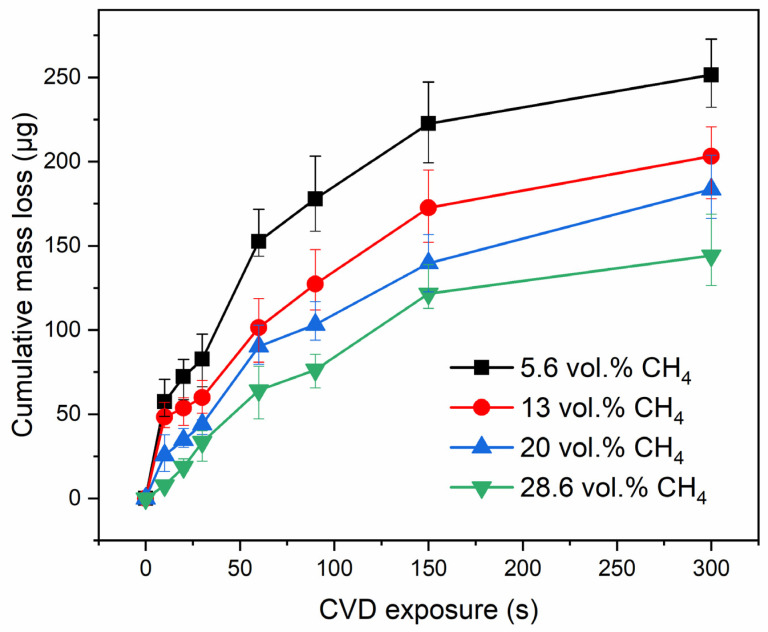
Plot of cumulative mass loss versus CVD operating time at various methane concentrations.

The CML of the samples was measured at various methane concentrations (Figure 6). The etching rate decreased with decreasing hydrogen concentration in the gas phase. Based on the CML data, it was calculated that an increase in the methane concentration by 10% decreases the CNTs etching rate by ~18%.

### 3.3. TEM Observations

**Figure 7:**
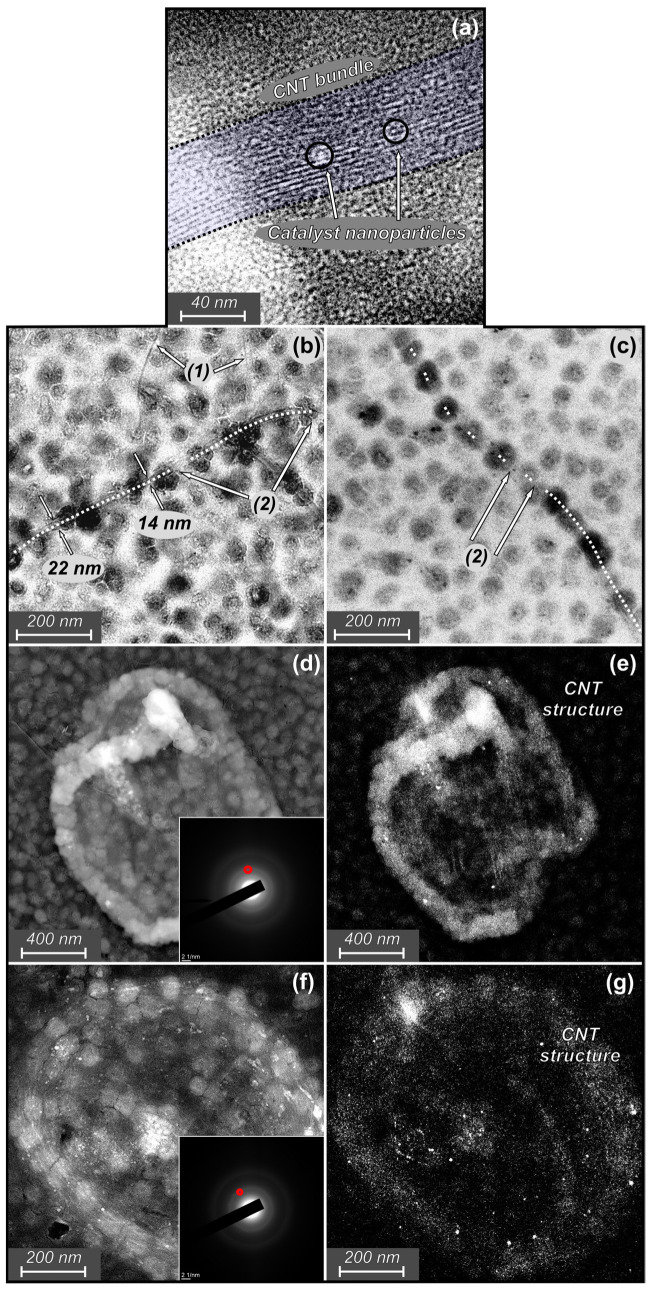
TEM images. BFTEM image of the SWCNTs bundle observed at 150 s CVD exposure (28.6 vol.% CH_4_) (**a**); BFTEM images of CNTs observed at 30 s (**b**) and 150 s (**c**) CVD exposure (28.6 vol.% CH_4_) that present CNTs bundles (dash line), SWCNTs (1), tube cuts (2) and bundle diameter measurements; BFTEM and SAED images (inserts) of HDAs observed at 30 s (**d**) and 150 s; (**f**) CVD exposure (28.6 vol.% CH_4_); and corresponding DFTEM images (**e**,**g**).

TEM analysis (Figure 7) was performed for the samples shown in Figure 5a,b, i.e., CVD treated at 28.6 vol.% CH_4_ for 30 and 150 s (silicon substrates).

Figure 7a shows a typical structure of a CNTs bundle containing SWCNTs; the same bundle structure was reported in other works [34,35]. In the sample in Figure 7b, both single nanotubes (Figure 7 (1)) and CNTs bundles (Figure 7 dash line) can be observed, and at 30 s a distinct crystal structure could still be traced in CNTs. It should be noted that single CNTs were observed on the sample treated for 30 s (Figure 7b), while on the sample treated for 150 s (Figure 7c) only CNTs bundles were presented. It is shown that thinning occurred unevenly along the length of the nanotubes. For the same CNTs bundle, the change in diameter was measured from 22 to 14 nm. Cutting (Figure 7 (2)) has been shown to be relatively rare at 30 s of CVD treatment. A change was observed after 150 s of CVD treatment (Figure 7c). It can be seen that the crystal structure of nanotubes becomes less distinct with frequent cuts along the nanotubes’ length. Additionally, a sufficient amount of CNTs material was removed by H-etching. The decrease in the intensity of CNT structures was observed with DFTEM (Figure 7e,g). Since the DFTEM images are acquired by selecting a certain reflex of the SAED pattern, we chose the reflections (Figure 7 red circles in SAED patterns inserts) on the ring characterizing the carbon nanotubes (graphene) structure, and observed that with an increase in the CVD treatment time, the intensity of the structures decreased, which indicated a violation of the structural integrity of the nanotubes and, therefore, their amorphization. The sharpness of the SAED rings corresponding to the CNTs also decreased, which additionally indicated a decrease in the crystallinity of the structure of the remaining nanotubes. Both the thinning and cutting of nanotubes are supported by the amorphization process.

### 3.4. Raman Observations

**Figure 8:**
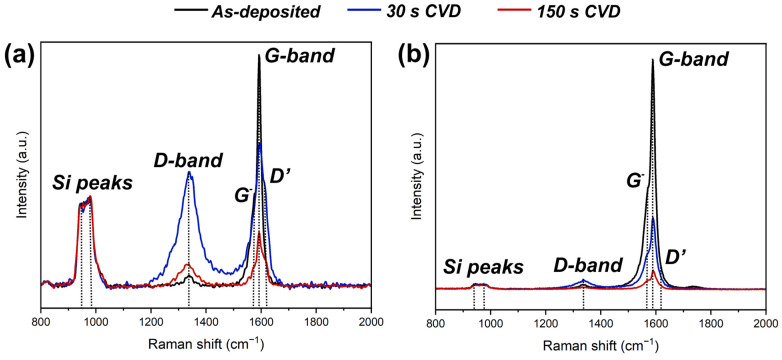
Typical Raman spectra of evenly distributed CNTs web (**a**) and HDA (**b**) taken from Raman mapping: black curves—as-deposited CNTs; blue curves—after 30 s of CVD (28.6 vol.% CH_4_); red curves—after 150 s of CVD (28.6 vol.% CH_4_).

To obtain statistical data on Raman spectra, we performed Raman mapping. Raman spectroscopy was carried out only for Si substrate samples. In the Raman spectra maps (not presented), two typical spectra were observed; the spectrum of an evenly distributed network of carbon nanotubes (Figure 8a), and the spectrum of a high-density agglomerations of nanotubes (Figure 8b).

Spectra of as-deposited CNTs layers are shown by black curves. The spectra show the second-order Raman peaks of silicon substrate (943 and 978 cm^−1^) [36]. They are shown for the purpose of comparing their intensity with the peaks of the deposited material. The peak of the CNTs structure consisted of a high-intensity G-band peak (1590 cm^−1^) and its left shoulder, i.e., the G^−^ peak at 1570 cm^−1^, which is characteristic of SWCNTs [37]. The G-band peak shape was the same in both the evenly-distributed CNTs web and HDA. There was also a low-intensity D-band peak at 1335 cm^−1^. This peak is associated with both the presence of amorphous carbon and with structural defects of nanotubes [37].

Blue curve spectra in Figure 8 corresponded to the sample presented in Figure 5a. The deposition of amorphous carbon on the surface of CNTs was observed as an increase in the D peak, and the ratio of I_d_/I_g_ (D-band peak intensity/G-band peak intensity) changed from 0.06 ± 0.02 to 0.81 ± 0.08 and from 0.02 ± 0.01 to 0.14 ± 0.02 for CNTs web and HDA, respectively. Despite the predominance of methyl radicals over atomic hydrogen in the gas mixture, which caused the deposition of amorphous carbon, Raman spectroscopy data indicated a decrease in the thickness of the CNTs/amorphous carbon layer, which can be seen from the decrease in the I_g_/I_si_ (G-band peak intensity/mean Si peaks intensity) ratio from 2.49 ± 0.7 to 1.67 ± 0.33 and from 38.56 ± 10.7 to 15.51 ± 6.98 for CNTs web and HDA, respectively. In addition, after 30 s of CVD treatment, a D’ peak (1620 cm^−1^) appeared near the G peak. The D’ peak is associated with defects in the graphitic crystal structure [38]. The red curve spectra in Figure 8 corresponded to the sample presented in Figure 5b. A further decrease in the thickness of the CNTs/amorphous carbon layer could be observed, which was characterized by a decrease in the I_g_/I_si_ ratio to 0.6 ± 0.11 and 5.1 ± 1.39 for CNTs web and HDA, respectively.

Based on the analysis of the I_d_/I_g_ ratios for samples 30 s and 150 s, we assume that the etching rate of amorphous carbon is higher than that of nanotubes. These ratios, coupled with the measured CML of carbon nanotubes, suggest the theoretical possibility of a parameters window in which hydrogen could selectively etch the amorphous phase formed during diamond synthesis, while preserving nanotubes. However, a further increase in the concentration of methane in the HFCVD method is limited by the fact that at high methane contents, the intensive carburization of hot tungsten filaments begins, which leads to their rapid destruction [39].

## 4. Conclusions

In this work, we studied the possibility of integrating preliminarily deposited CNTs into the CVD diamond synthesis process and the dynamics of processes occurring with nanotubes under these conditions. In the entire studied range of parameters, intense hydrogen etching of CNTs was observed, which led to the complete disappearance of the CNTs layer from the substrate surface before the diamond growth process began. This indicates the impossibility of synthesizing a diamond-CNT composite under the studied conditions. It was calculated that with a decrease in the hydrogen concentration by 10 vol.%, the etching rate of CNTs would decrease by ~18%. Two main mechanisms of CNTs destruction are shown—thinning and cutting, which were accompanied by amorphization. Thinning is the main mechanism of etching of small-diameter CNTs bundles. Cutting is mostly found in thicker bundles, where it starts at defect sites and then propagates through the length of a tube. Based on the results of CML and Raman analysis, we hypothesize that it may be possible to achieve a parameter window in which the H-etching rate is reduced just enough to etch the amorphous carbon and preserve the CNTs. However, the HFCVD method is limited by a maximum methane concentration. We suggest two possible solutions for the problem of hydrogen etching. The first is a change in the gas composition to a hydrogen-free mixture, for example CH_4_/Ar. The second is a change in the gas activation system from hot filaments to microwave plasma or arc discharge, as it will allow the use of higher methane concentrations.

## Figures and Tables

**Table 1:** Deposition parameters used for simultaneous diamond–CNTs composite growth.

№	Method	Working Gas Composition	Substrate Temperature, °C	Pressure, Torr	Catalyst	Reference
1	HFCVD	CH_4_/H_2_ (96.9–99.4% H_2_)	750	20–30	Ni	[21]
2	HFCVD	CH_4_/H_2_ (95.67–97.34% H_2_)	~800	50	Fe~70% Cr~20% Ni~10%	[22]
3	MPCVD	CH_4_/H_2_ (84.7% H_2_)	>700	75	Cast iron	[23]
4	MPCVD	Ar/CH_4_ (99% Ar)	700	15	Fe	[24]
5	HFCVD	CH_4_/H_2_ (99.7% H_2_)	500	20	Fe	[25]

**Table 2:** Peak intensity ratios derived from Raman spectra.

CVD Time, Sec	Evenly Distributed CNTs		HDA	
	I_d_/I_g_	I_g_/I_si_	I_d_/I_g_	I_g_/I_si_
0	0.06 ± 0.02	2.49 ± 0.7	0.02 ± 0.01	38.56 ± 10.7
30	0.81 ± 0.08	1.67 ± 0.33	0.14 ± 0.02	15.51 ± 6.98
150	0.42 ± 0.09	0.6 ± 0.11	0.11 ± 0.06	5.1 ± 1.39

## Data Availability

Data sharing is not applicable to this article.

## References

[1] Koziol K., Vilatela J., Moisala A., Motta M., Cunniff P., Sennett M., Windle A. (2007). High-Performance Carbon Nanotube Fiber. Science.

[2] Chou T.W., Gao L., Thostenson E.T., Zhang Z., Byun J.H. (2010). An Assessment of the Science and Technology of Carbon Nanotube-Based Fibers and Composites. Compos. Sci. Technol..

[3] Jolowsky C., Sweat R., Park J.G., Hao A., Liang R. (2018). Microstructure Evolution and Self-Assembling of CNT Networks during Mechanical Stretching and Mechanical Properties of Highly Aligned CNT Composites. Compos. Sci. Technol..

[4] Berber S., Kwon Y.K., Tománek D. (2000). Unusually High Thermal Conductivity of Carbon Nanotubes. Phys. Rev. Lett..

[5] Qiu L., Zhu N., Feng Y., Michaelides E.E., Żyła G., Jing D., Zhang X., Norris P.M., Markides C.N., Mahian O. (2020). A Review of Recent Advances in Thermophysical Properties at the Nanoscale: From Solid State to Colloids. Phys. Rep..

[6] Park J.G., Smithyman J., Lin C.Y., Cooke A., Kismarahardja A.W., Li S., Liang R., Brooks J.S., Zhang C., Wang B. (2009). Effects of Surfactants and Alignment on the Physical Properties of Single-Walled Carbon Nanotube Buckypaper. J. Appl. Phys..

[7] Yang L., Li S., Zhou X., Liu J., Li Y., Yang M., Yuan Q., Zhang W. (2019). Effects of Carbon Nanotube on the Thermal, Mechanical, and Electrical Properties of PLA/CNT Printed Parts in the FDM Process. Synth. Met..

[8] Mikhalchan A., Vilatela J.J. (2019). A Perspective on High-Performance CNT Fibres for Structural Composites. Carbon.

[9] Nurazzi N.M., Asyraf M.R.M., Khalina A., Abdullah N., Sabaruddin F.A., Kamarudin S.H., Ahmad S., Mahat A.M., Lee C.L., Aisyah H.A. (2021). Fabrication, Functionalization, and Application of Carbon Nanotube-Reinforced Polymer Composite: An Overview. Polymers.

[10] Mostafa A.M., Mwafy E.A., Toghan A. (2021). ZnO Nanoparticles Decorated Carbon Nanotubes via Pulsed Laser Ablation Method for Degradation of Methylene Blue Dyes. Colloids. Surf. A Physicochem. Eng. Asp..

[11] Ribeiro H., Schnitzler M.C., da Silva W.M., Santos A.P. (2021). Purification of Carbon Nanotubes Produced by the Electric Arc-Discharge Method. Surf. Interfaces.

[12] Manawi Y.M., Ihsanullah, Samara A., Al-Ansari T., Atieh M.A. (2018). A Review of Carbon Nanomaterials’ Synthesis via the Chemical Vapor Deposition (CVD) Method. Materials.

[13] Wang X.-D., Vinodgopal K., Dai G.-P. (2019). Synthesis of Carbon Nanotubes by Catalytic Chemical Vapor Deposition. Perspective of Carbon Nanotubes.

[14] Varanasi C., Petry J., Brunke L., Yang B.T., Lanter W., Burke J., Wang H., Bulmer J.S., Scofield J., Barnes P.N. (2010). Growth of High-Quality Carbon Nanotubes on Free-Standing Diamond Substrates. Carbon.

[15] Tsai P.H., Tsai H.Y. (2015). Fabrication and Field Emission Characteristic of Microcrystalline Diamond/Carbon Nanotube Double-Layered Pyramid Arrays. Thin Solid Film..

[16] Zhao G., Li Z., Hu M., Li L., He N., Jamil M. (2019). Fabrication and Performance of CVD Diamond Cutting Tool in Micro Milling of Oxygen-Free Copper. Diam. Relat. Mater..

[17] Almeida F.A., Sacramento J., Oliveira F.J., Silva R.F. (2008). Micro- and Nano-Crystalline CVD Diamond Coated Tools in the Turning of EDM Graphite. Surf. Coat. Technol..

[18] Gracio J.J., Fan Q.H., Madaleno J.C. (2010). Diamond Growth by Chemical Vapour Deposition. J. Phys. D Appl. Phys..

[19] Sedov V.S., Martyanov A.K., Khomich A.A., Savin S.S., Zavedeev E.V., Ralchenko V.G. (2020). Deposition of Diamond Films on Si by Microwave Plasma CVD in Varied CH_4_-H_2_ Mixtures: Reverse Nanocrystalline-to-Microcrystalline Structure Transition at Very High Methane Concentrations. Diam. Relat. Mater..

[20] Gaydaychuk A., Linnik S., Mitulinsky A., Zenkin S., Bulakh V. (2023). Comparative Analysis of Working Gas Composition Impact on Diamond Films Microstructure. Surf. Interfaces.

[21] Yang Q., Xiao C., Chen W., Hirose A. (2004). Selective Growth of Diamond and Carbon Nanostructures by Hot Filament Chemical Vapor Deposition. Diam. Relat. Mater..

[22] Kumaran C.R., Chandran M., Krishna Surendra M., Bhattacharya S.S., Ramachandra Rao M.S. (2015). Growth and Characterization of Diamond Particles, Diamond Films, and CNT-Diamond Composite Films Deposited Simultaneously by Hot Filament CVD. J. Mater. Sci..

[23] Fernandes A.J.S., Pinto M., Neto M.A., Oliveira F.J., Silva R.F., Costa F.M. (2009). Nano Carbon Hybrids from the Simultaneous Synthesis of CNT/NCD by MPCVD. Diam. Relat. Mater..

[24] Xiao X., Elam J.W., Trasobares S., Auciello O., Carlisle J.A. (2005). Synthesis of a Self-Assembled Hybrid of Ultrananocrystalline Diamond and Carbon Nanotubes. Adv. Mater..

[25] Varshney D., Weiner B.R., Morell G. (2010). Growth and Field Emission Study of a Monolithic Carbon Nanotube/Diamond Composite. Carbon.

[26] Guglielmotti V., Chieppa S., Orlanducci S., Tamburri E., Toschi F., Terranova M.L., Rossi M. (2009). Carbon Nanotube/Nanodiamond Structures: An Innovative Concept for Stable and Ready-to-Start Electron Emitters. Appl. Phys. Lett..

[27] Sun Y., Wu J., He L., Liu B., Zhang C., Meng Q., Zhang X. (2020). Influence of B4C Coating on Graphitization for Diamond/WC-Fe-Ni Composite. Int. J. Refract. Met. Hard Mater..

[28] Haubner R., Schubert W.D., Lux B. (1998). Interactions of Hard Metal Substrates during Diamond Deposition. Int. J. Refract. Met. Hard Mater..

[29] Shankar N., Glumac N.G., Yu M.F., Vanka S.P. (2008). Growth of Nanodiamond/Carbon-Nanotube Composites with Hot Filament Chemical Vapor Deposition. Diam. Relat. Mater..

[30] Rao F.B., Li T., Wang Y.L. (2008). Effect of Hydrogen on the Growth of Single-Walled Carbon Nanotubes by Thermal Chemical Vapor Deposition. Phys. E Low Dimens. Syst. Nanostructures.

[31] Zhang G., Qi P., Wang X., Lu Y., Mann D., Li X., Dai H. (2006). Hydrogenation and Hydro-Carbonation and Etching of Single-Walled Carbon Nanotubes. J. Am. Chem. Soc..

[32] Kobayashi K., Nakano T., Mutsukura N., Machi Y. (1993). Characterization of Diamond Nucleation on Fe/Si Substrate by Hot-Filament Chemical Vapor Deposition. Vacuum.

[33] Louchev O.A., Dussarrat C., Sato Y. (1999). Surface Carbonization and Nucleation during Chemical Vapor Deposition of Diamond. J. Appl. Phys..

[34] Lisowski W., Keim E.G., Van Den Berg A.H.J., Smithers M.A. (2005). Structural and Chemical Evolution of Single-Wall Carbon Nanotubes under Atomic and Molecular Deuterium Interaction. Carbon.

[35] Predtechenskiy M.R., Khasin A.A., Bezrodny A.E., Bobrenok O.F., Dubov D.Y., Muradyan V.E., Saik V.O., Smirnov S.N. (2022). New Perspectives in SWCNT Applications: Tuball SWCNTs. Part 1. Tuball by Itself—All You Need to Know about It. Carbon Trends.

[36] Gillet Y., Kontur S., Giantomassi M., Draxl C., Gonze X. (2017). Ab Initio Approach to Second-Order Resonant Raman Scattering Including Exciton-Phonon Interaction. Sci. Rep..

[37] Park Y., Hembram K.P.S.S., Yoo R., Jang B., Lee W., Lee S.G., Kim J.G., Kim Y., Moon D.J., Lee J.K. (2019). Reinterpretation of Single-Wall Carbon Nanotubes by Raman Spectroscopy. J. Phys. Chem. C.

[38] Wu J., Bin, Lin M.L., Cong X., Liu H.N., Tan P.H. (2018). Raman Spectroscopy of Graphene-Based Materials and Its Applications in Related Devices. Chem. Soc. Rev..

[39] Kromka A., Babchenko O., Potocky S., Rezek B., Sveshnikov A., Demo P., Izak T., Varga M., Narayan R. (2013). Diamond Nucleation and Seeding Techniques for Tissue Regeneration. Diamond-Based Materials for Biomedical Applications.

